# Extensively Drug-Resistant Klebsiella pneumoniae Counteracts Fitness and Virulence Costs That Accompanied Ceftazidime-Avibactam Resistance Acquisition

**DOI:** 10.1128/spectrum.00148-22

**Published:** 2022-04-18

**Authors:** Elias Eger, Michael Schwabe, Lukas Schulig, Nils-Olaf Hübner, Jürgen A. Bohnert, Uwe T. Bornscheuer, Stefan E. Heiden, Justus U. Müller, Fazal Adnan, Karsten Becker, Carlos L. Correa-Martinez, Sebastian Guenther, Evgeny A. Idelevich, Daniel Baecker, Katharina Schaufler

**Affiliations:** a Pharmaceutical Microbiology, Institute of Pharmacy, University of Greifswald, Greifswald, Germany; b Pharmaceutical and Medicinal Chemistry, Institute of Pharmacy, University of Greifswald, Greifswald, Germany; c Central Unit for Infection Prevention and Control, University Medicine Greifswald, Greifswald, Germany; d Friedrich Loeffler-Institute of Medical Microbiology, University Medicine Greifswald, Greifswald, Germany; e Biotechnology and Enzyme Catalysis, Institute of Biochemistry, University of Greifswald, Greifswald, Germany; f Atta-ur-Rahman School of Applied Biosciences, National University of Sciences and Technology, Islamabad, Pakistan; g Institute of Hygiene, University Hospital Münster, Münster, Germany; h Pharmaceutical Biology, Institute of Pharmacy, University of Greifswald, Greifswald, Germany; i Institute of Medical Microbiology, University Hospital Münster, Münster, Germany; j Institute for Infectious Diseases, Christian Albrecht University and Schleswig-Holstein University Medical Center Kiel, Kiel, Germany; Indian Institute of Science Bangalore

**Keywords:** XDR, ST307, OmpK36, experimental evolution, fitness and virulence compensation, RpoE

## Abstract

The ability of extensively drug-resistant (XDR) Klebsiella pneumoniae to rapidly acquire resistance to novel antibiotics is a global concern. Moreover, Klebsiella clonal lineages that successfully combine resistance and hypervirulence have increasingly occurred during the last years. However, the underlying mechanisms of counteracting fitness costs that accompany antibiotic resistance acquisition remain largely unexplored. Here, we investigated whether and how an XDR sequence type (ST)307 K. pneumoniae strain developed resistance against the novel drug combination ceftazidime-avibactam (CAZ-AVI) using experimental evolution. In addition, we performed *in vitro* and *in vivo* assays, molecular modeling, and bioinformatics to identify resistance-conferring processes and explore the resulting decrease in fitness and virulence. The subsequent amelioration of the initial costs was also addressed. We demonstrate that distinct mutations of the major nonselective porin OmpK36 caused CAZ-AVI resistance that persists even upon following a second experimental evolution without antibiotic selection pressure and that the Klebsiella strain compensates the resulting fitness and virulence costs. Furthermore, the genomic and transcriptomic analyses suggest the envelope stress response regulator *rpoE* and associated RpoE-regulated genes as drivers of this compensation. This study verifies the crucial role of OmpK36 in CAZ-AVI resistance and shows the rapid adaptation of a bacterial pathogen to compensate fitness- and virulence-associated resistance costs, which possibly contributes to the emergence of successful clonal lineages.

**IMPORTANCE** Extensively drug-resistant Klebsiella pneumoniae causing major outbreaks and severe infections has become a significant challenge for health care systems worldwide. Rapid resistance development against last-resort therapeutics like ceftazidime-avibactam is a significant driver for the accelerated emergence of such pathogens. Therefore, it is crucial to understand what exactly mediates rapid resistance acquisition and how bacterial pathogens counteract accompanying fitness and virulence costs. By combining bioinformatics with *in vitro* and *in vivo* phenotypic approaches, this study revealed the critical role of mutations in a particular porin channel in ceftazidime-avibactam resistance development and a major metabolic regulator for ameliorating fitness and virulence costs. These results highlight underlying mechanisms and contribute to the understanding of factors important for the emergence of successful bacterial pathogens.

## INTRODUCTION

Challenged by the increasing occurrence of extensively drug-resistant (XDR) (1–3) and even pan-drug-resistant (PDR) (4–6) Klebsiella pneumoniae strains, which are nonsusceptible to almost all classes of antibiotics (7), reliable treatment of health care- and community-associated infections caused by these pathogens poses a serious concern to the physician. The World Health Organization (WHO) ranked carbapenem-resistant K. pneumoniae as one of the most critical priority pathogens. This highlights the need for the development of novel antibiotics and treatment options (8). One recent example for the latter is the fixed-dose drug combination ceftazidime-avibactam (CAZ-AVI; ratio of 4:1). The United States Food and Drug Administration (FDA) approved and introduced CAZ-AVI on the market in 2015, followed by the European Medicines Agency (EMA) 1 year later (9).

While ceftazidime (CAZ) is an established third-generation cephalosporin, avibactam (AVI) is the first approved non-β-lactam β-lactamase inhibitor (10). AVI possesses potent activity against β-lactamases belonging to Ambler classes A (extended-spectrum β-lactamases [ESBL]; e.g., CTX-M-15 and K. pneumoniae carbapenemases [KPC]), C (AmpC β-lactamases), and D (oxacillinases; e.g., OXA-48). However, like other serine-acylating β-lactamase inhibitors, AVI cannot inhibit metallo-β-lactamases (MBL; e.g., NDM-1, Ambler class B) (11). Therefore, treating acute and life-threatening infections caused by XDR K. pneumoniae strains positive for both MBL and non-MBL (such as CTX-M-15 and OXA-48) by applying CAZ-AVI in combination with the only clinically available monobactam aztreonam (ATM), which is not inactivated by MBL (12), might represent the last possible therapy option. This combination was used, for example, to treat an outbreak caused by sequence type (ST)307 K. pneumoniae in different health care institutions in Western Pomerania (Germany) (1, 13) and an outbreak by ST147 K. pneumoniae in a hospital in Barcelona (Spain) (14). Studies have previously reported CAZ-AVI and ATM-AVI resistances in *Enterobacterales*. The respective strains showed decreased membrane permeability through changes of outer membrane proteins (OMPs) (15), induction of efflux (16), modification of the targeted penicillin-binding protein 3 (PBP3) (17), or point mutations in active sites of β-lactamases (e.g., Lys237Gln of CTX-M-15 [18], Pro68Ala in combination with Tyr211Ser of OXA-48 [19], and Ala172Thr of KPC-3 [20]). The broad genetic diversity and range of known and unknown mechanisms of antimicrobial resistance acquisition underline the continued need for the further and in-depth characterization of resistance determinants.

In addition to “classical” carbapenem-resistant K. pneumoniae (cKp) described above, there is the hypervirulent K. pneumoniae (hvKp) pathotype (21–23). The latter is defined by its general susceptibility to antibiotics, the community association of infections caused, and high-level virulence, which is characterized phenotypically by hypermucoviscosity and extensive siderophore secretion (24–27). Clinically, infections caused by hvKp are often invasive and include, for instance, pyogenic liver abscess, pneumonia, endophthalmitis, meningitis, necrotizing fasciitis, and bacteremia (22). Alarmingly, the convergence of both cKp and hvKp has already been frequently described (1, 2, 28), which blurs the boundaries between the pathotypes. This phenomenon is seemingly driven not only by the exchange of resistance (29–31) and virulence plasmids (28, 32) but also by the chromosomal integration of DNA sequences with uncommonly high length (23, 33–35) as well as rearranged “mosaic” plasmids that carry both resistance- and virulence-associated genes (1, 36–38). We have previously reported on such mosaic plasmids, which do not necessarily reduce bacterial fitness, in the above-mentioned ST307 K. pneumoniae clonal lineage (1). Thus, this apparent ability of bacterial pathogens to improve their fitness through plasmid adaptation and compensatory mutations might explain the success of some clonal lineages (39). However, due to the redundancy and epistasis of genes and complexities of underlying pathways (40, 41), little is known about how and which mutations and regulatory mechanisms counteract fitness reduction (and associated virulence) caused by antimicrobial resistance acquisition (39).

Here, we investigated (i) the acquisition of resistance against CAZ-AVI as well as cooccurring resistances of a clinical K. pneumoniae strain and (ii) underlying mechanisms following an experimental evolution (EE) approach. Then, we explored (iii) whether and how this resistance acquisition might be compensated, again based on EE and downstream analyses. Overall, by combining a comprehensive set of *in vitro* and *in vivo* experiments with genomic and transcriptomic analyses, we reveal deep insights into the resistance evolution against CAZ-AVI and accompanied compensatory mechanisms to ameliorate fitness and virulence costs in XDR K. pneumoniae.

## RESULTS

### Increased tolerance against CAZ-AVI and ATM due to reduced membrane permeability.

The emergence of XDR K. pneumoniae has been frequently described (1–3). However, we do not yet fully understand the underlying mechanisms and dynamics of resistance acquisition, especially against newer antibiotics and drug combinations. One objective of this study was thus to investigate resistance development against CAZ-AVI. We used a well-characterized XDR, yet CAZ-AVI-sensitive, ST307 K. pneumoniae strain (PBIO2003) (1) obtained from the outbreak mentioned earlier that took place in four different health care institutions in Western Pomerania (Germany) in 2019 and the beginning of 2020. Compared to the other ST307 isolates from the outbreak, PBIO2003 had lost the *bla*_NDM-1_ gene and, therefore, showed susceptibility to CAZ-AVI and CAZ-AVI combined with ATM.

**FIG 1:**
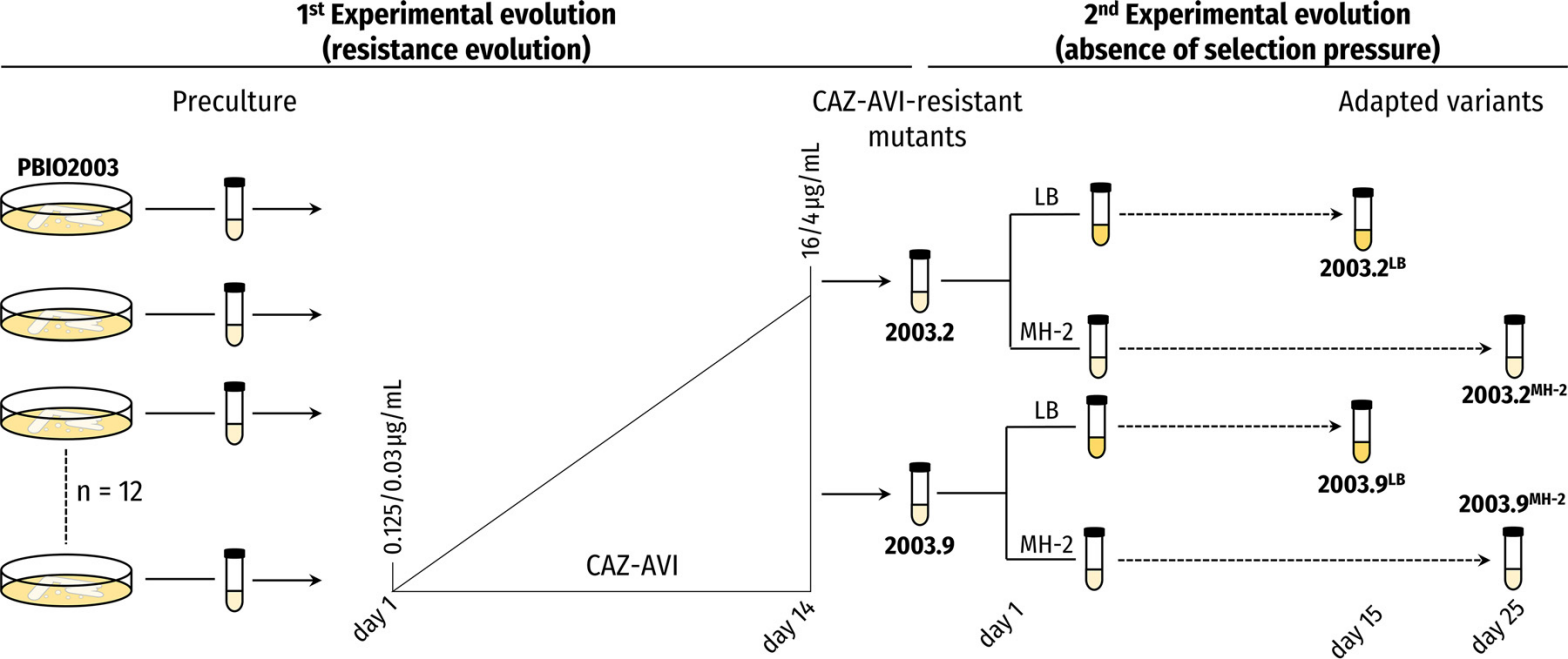
Schematic presentation of the experimental design. To investigate the resistance acquisition against CAZ-AVI, we inoculated 12 randomly chosen single colonies of PBIO2003 individually in 1 mL of MH-2 and incubated them overnight (preculture). Then, the stationary-phase cultures were transferred daily in the presence of increasing CAZ-AVI concentrations until some replicates tolerated concentrations of 16/4 μg/mL CAZ-AVI. We then used a second EE approach to investigate compensatory events overcoming (putative) fitness burdens. One population, each of 2003.2 and 2003.9, was propagated in LB or MH-2, and an everyday stationary-phase culture was transferred into fresh medium.

Originally, we started with 12 biological replicates and a concentration of 0.125/0.03 μg/mL CAZ-AVI (one-fourth MIC; Fig. 1). With daily increasing concentrations of up to 16/4 μg/mL CAZ-AVI (which is considered resistant according to the European Committee on Antimicrobial Susceptibility Testing [EUCAST] guidelines [42]), we obtained overall two resistant replicates (16.67%, 2/12) within 14 days.

**FIG 2:**
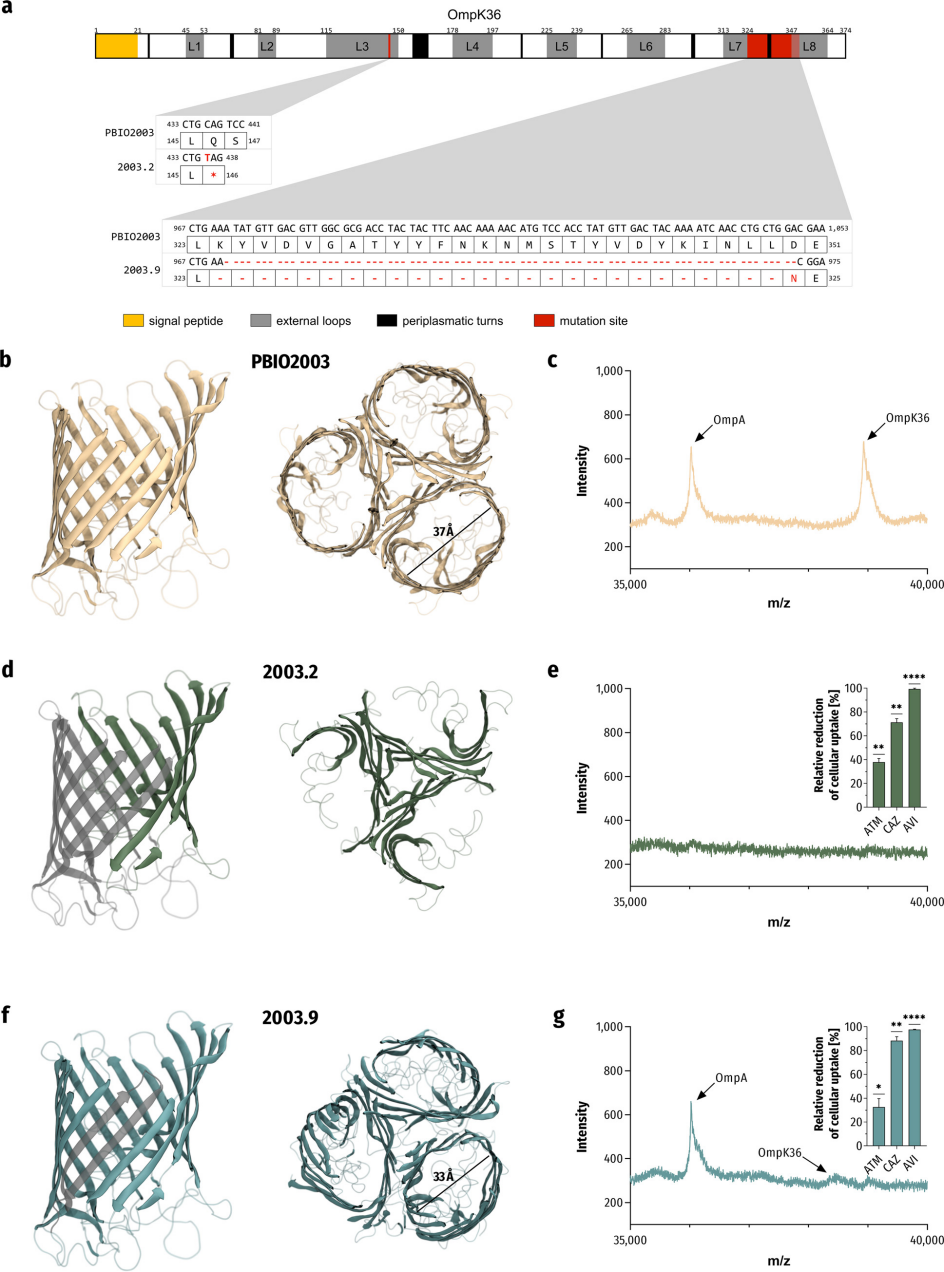
Different mutations of *ompK36* change the outer membrane proteins’ architecture and reduce CAZ, AVI, and ATM uptake. (a) Schematic presentation of genetic changes in the *ompK36* gene of the CAZ-AVI-resistant mutants 2003.2 and 2003.9. The different mutations are marked at their respective positions (red). (b, d, and f) Cartoon representation of modeled protein structures of the trimeric OmpK36 of PBIO2003 (wild-type; b), 2003.2 (premature stop codon; d), and 2003.9 (deletion of β-sheets between the external loops L7 and L8; f). Predicted changes in the architecture of the porin channel in lateral view (left) are colored in transparent gray. (c, e, and g) Mass spectra of outer membrane proteins represent differences in the configuration of expressed proteins of 2003.2 (e) and 2003.9 (g) compared to PBIO2003 (c). The insets show that changes in membrane permeability reduce CAZ, AVI, and ATM uptake into 2003.2 and 2003.9. The results are given as mean values of percent relative reduction related to PBIO2003 and standard error (*n* = 3). The results were analyzed using a one-sample *t* test, and the following indicate the significance level (*P* value): *, *P* < 0.05; **, *P* < 0.01; ****, *P* < 0.0001.

When comparing the CAZ-AVI-resistant mutants (subsequently termed 2003.2 and 2003.9) with the genome of the ancestral wild-type strain (PBIO2003), our analysis revealed different mutations in the *ompK36* gene (Fig. 2). This gene encodes an OMP (designated porin) orthologue of Escherichia coli OmpC (43). Porins are β-barrel proteins composed of antiparallel β-sheets, thus forming an essential aqueous transmembrane channel system (44). For 2003.2, we found a nonsense single-nucleotide polymorphism (SNP) of *ompK36* (436C>T [Gln146*]; Fig. 2a), resulting in a premature stop codon and ultimately truncated translation, which usually leads to a nonfunctional protein. To further explore the molecular effects of this mutation, we predicted the protein structure of OmpK36 by homology modeling (Fig. 2b, d, and f). As shown in Fig. 2d, the protein structure model of the premature stop codon mutation supported our prediction regarding loss of function due to the absence of large parts of the porin channel transmembrane region. Additionally, we identified a read-through mutation in *dsbA* (624A>T [*208Tyr]) with a putative in-frame C terminus extension of 16 amino acids in 2003.2 that may lead to changes in protein expression levels and stability (45). This gene encodes a thiol-disulfide interchange protein, typically required for disulfide bond formation in periplasmatic proteins such as OmpA (46, 47). The second mutant (2003.9) showed a fragment deletion 78 bp in length (corresponding to 26 codons), resulting in an intact reading frame with a predicted deletion of transmembrane β-sheets between the external loops L7 and L8 and a reduced pore diameter (Fig. 2b and f). Note, however, that the structure generated for 2003.9 does not represent a complete atomistic model but is instead used to visualize a significant decrease of the β-barrel diameter, explaining the possible resistance mechanism.

We then used a matrix-assisted laser desorption ionization–time of flight mass spectrometry (MALDI-TOF MS) approach to identify the expressed outer membrane proteins and evaluate our predicted phenotypes (Fig. 2c, e, and g). Mutant 2003.2 (premature stop codon mutation; Fig. 2e) revealed a lack of the OmpK36 signal (*m*/*z* of 38,933). Interestingly, we also obtained no signal associated with the OmpA channel (*m*/*z* of 36,026). The β-strand-deleted variant (2003.9; Fig. 2g) showed a shift of the OmpK36 signal based on mass loss (*m*/*z* of 38,465). Notably, both the wild-type strain PBIO2003 (Fig. 2c) and mutants (2003.2 and 2003.9) showed only a low coverage of the *ompK35* annotation. This was consistent with our findings in the MALDI-TOF MS examination of the outer membrane proteins (*m*/*z*_expected_ of 37,926).

It is well known that cross-resistance (48) and collateral sensitivity (49) may accompany resistance acquisition. To further investigate the effect of the aforementioned mutations on the antibiotic resistance profile, we determined MICs of a large panel of relevant antibiotic drugs and combinations (Table S1 in the supplemental material). Phenotypic antimicrobial susceptibility testing (AST) revealed for 2003.2 and 2003.9 a strong increase not only of CAZ-AVI MIC values (>64/4 μg/mL CAZ-AVI) but also of CAZ-AVI in combination with ATM (>64/4/64 μg/mL CAZ-AVI/ATM). Interestingly, we found no evidence of collateral sensitivity as described previously in another study (50). We then analyzed potential differences in the compound uptake of each of the triple combination of the β-lactam antibiotics ATM and CAZ and the non-β-lactam β-lactamase inhibitor AVI. Therefore, we used a high-resolution continuum-source molecular absorption spectrometry (HR CS MAS) approach to determine changes in endogenous sulfur content following treatment with the sulfurous compounds (Fig. 2e and g). Both mutants showed similar trends of relative uptake reductions compared to PBIO2003, whereby the uptake of CAZ and AVI seemed highly affected by the reduced outer membrane permeability. However, while the uptake of CAZ was significantly reduced by up to 71.3% (PBIO2003 versus 2003.2: *P* = 0.0019) and 88.4% (PBIO2003 versus 2003.9: *P* = 0.0014), respectively, ATM uptake was reduced by only about one-third (PBIO2003 versus 2003.2: 37.9%, *P* = 0.0012; PBIO2003 versus 2003.9: 32.6%, *P* = 0.0450). The uptake of AVI was almost no longer measurable (PBIO2003 versus 2003.2: 99.3%, *P* < 0.0001; PBIO2003 versus 2003.9: 97.6%, *P* < 0.0001), thus supporting the role of OmpK36 in resistance acquisition against CAZ-AVI and ATM.

In summary, our results suggest that the decreased membrane permeability through mutation of the *ompK36* gene conferred resistance against CAZ-AVI and CAZ-AVI/ATM. Moreover, similar changes in MICs and comparable reduction in compound uptake indicate that the expression of OmpK36 with reduced pore diameter (2003.9) is as effective as the complete deletion of the porin (2003.2). In addition, we show that the uptake of the bridged 1,6-diazabicyclo[3.2.1]octan-7-one derivative AVI is highly dependent on OmpK36, which may suggest cross-resistance to other members of this class of diazabicyclooctanes β-lactamase inhibitors (e.g., nacubactam or zidebactam).

### Membrane impermeability negatively affects growth, virulence, resilience, and mortality.

**FIG 3:**
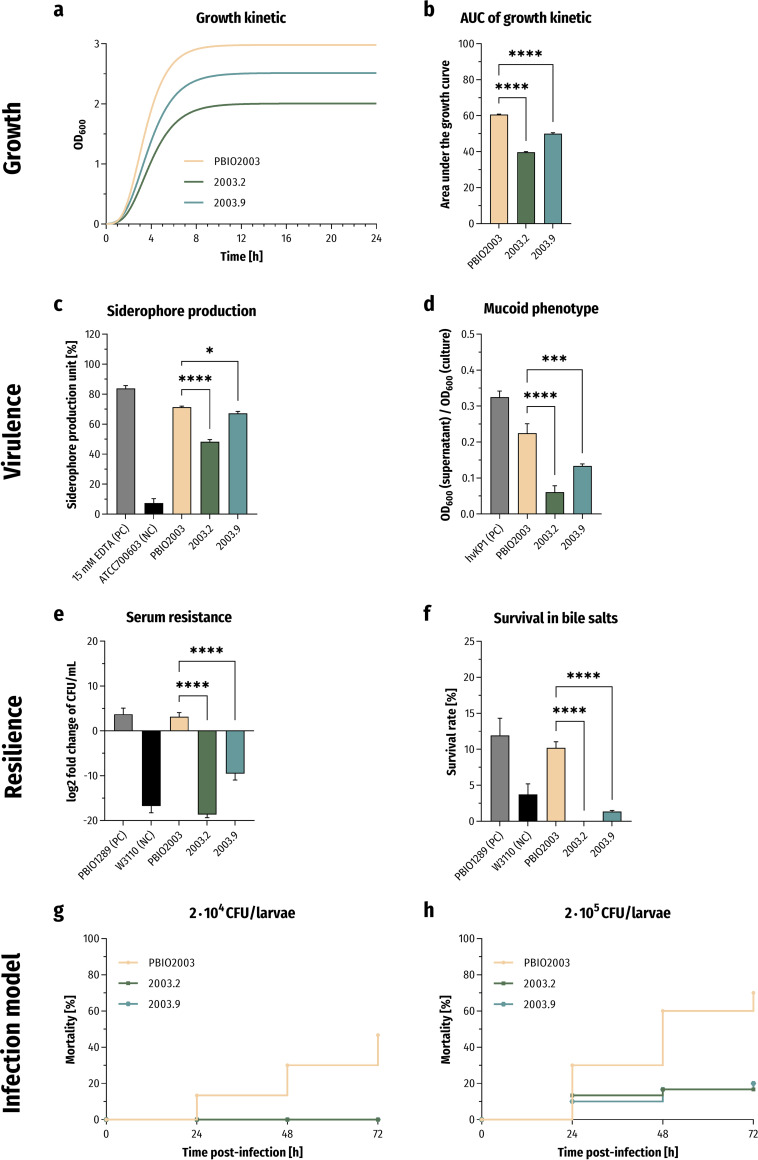
Phenotypic characteristics of the CAZ-AVI-resistant mutants 2003.2 and 2003.9. (a and b) Gompertz growth-fitting curves of the growth kinetics in LB (*n* = 3; a) and statistical comparison of area under the growth curves (AUC; b). The results are given as mean values and standard deviation of AUCs. (c) The extent of secreted siderophore is presented as mean values of the siderophore production unit and standard deviation (*n* = 3). (d) Determination of mucoid phenotype using a sedimentation assay (*n* = 3). The results are given as mean ratios of OD_600_ of supernatant after centrifugation at 1,000 × *g* for 5 min and total OD_600_ and standard deviation. (e) Survival in 50% human serum (*n* = 3). The results are given as mean values and standard deviation of log_2_ fold change of CFU/mL after 4 h of incubation in the presence of serum. (f) Resilience against 50 mg/mL bile salts (*n* = 3). The results are shown as mean percent survival rates and standard deviation. (g and h) Kaplan-Meier plot of mortality rates in the Galleria mellonella larvae infection model (*n* = 3). The results are given as mean percent mortality following injection of 2 × 10^4^ CFU/larvae (g) and 2 × 10^5^ CFU/larvae (h). For all results, the mutants were compared to PBIO2003 using variance analyses (one-way ANOVA with Dunnett’s multiple comparison *post hoc* test); *, *P* < 0.05; ***, *P* < 0.001; ****, *P* < 0.0001.

Although porin changes conferring reduced membrane permeability have been frequently described, those mutants rarely cause outbreaks (18). This might be explained by the fact that modification of nonspecific porins leads to a fitness reduction and/or, subsequently, a decrease of virulence (51–55). This is why we challenged the porin mutants 2003.2 and 2003.9 in phenotypic experiments, including growth, virulence, resilience, and *in vivo* mortality, to investigate whether these mutations influence clinically relevant features important for bacterial pathogenesis (Fig. 3).

We observed significantly decreased growth behaviors in LB for both mutants compared to wild-type PBIO2003 (area under the growth curves [AUC] of 60.61; Fig. 3a and b), with 2003.2 (AUC of 39.72, *P* < 0.0001) showing a higher decrease than 2003.9 (AUC of 50.08, *P* < 0.0001).

We next determined siderophore secretion and mucoid phenotypes to investigate whether the growth decrease was associated with a lower virulence level (Fig. 3c and d). The biosynthesis and secretion of siderophores, which are small iron-chelating compounds that seize iron from the host (56), play a crucial role during bacterial infection and are hallmarks of hypervirulent K. pneumoniae (57). Our analysis revealed a significant decrease of siderophore secretion for 2003.2 (48.3%, *P* < 0.0001) and 2003.9 (67.3%, *P* = 0.0102) compared to PBIO2003 (71.3%; Fig. 3c). Another important virulence-associated feature of pathogenic K. pneumoniae is capsule formation, which functions as a physiological barrier and conveys protection against the hosts’ immune system (e.g., protection from phagocytosis) (58, 59). Hypermucoviscosity quantification is based on prolonged sedimentation and the following retaining of mucus in the supernatant after centrifugation of hypermucoviscous cells (60). Here, the mutant cells almost entirely sedimented and formed a tighter pellet than the wild-type cells (PBIO2003: 0.225), thus indicating decreased mucoviscosity (Fig. 3d). Again, we noticed a higher decrease in this virulence-associated feature in 2003.2 (0.061, *P* < 0.0001) than in 2003.9 (0.134, *P* = 0.0001).

Since K. pneumoniae is a leading cause of pyogenic liver abscesses (61–64) and bacteremia (65, 66), its resilience against bile salts and serum seems essential for pathogenesis. Therefore, and to investigate the impact of reduced membrane permeability on bacterial stress response and resilience, we challenged the mutants with complement-containing human serum and bile salts for 4 h each. We observed a significant decrease in survival of the OmpK36 mutants in response to both external stressors, which indicates reduced pathogenicity of 2003.2 and 2003.9 compared to PBIO2003 (Fig. 3e and f).

We finally performed a larvae infection model of the greater wax moth Galleria mellonella to study the mutants’ ability to cause infection-associated mortality *in vivo* (67). We injected equivalent numbers of CFU of the wild-type and both OmpK36 mutants in the right proleg and monitored the death of larvae every 24 h. When injecting 2 × 10^4^ CFU of PBIO2003 per larvae, we detected a mortality rate of 13.3% after 24 h and 46.7% after 72 h of incubation (Fig. 3g). When using the same inoculation size of 2003.2 and 2003.9, we observed 100% larval survival 72 h after injection. Mortality rates increased slightly when higher concentrations of the mutants (2 × 10^5^ CFU) were used, but they remained significantly lower than for the wild-type (Fig. 3h).

### Adaptative mechanisms increase fitness, virulence, and resilience while resistance is retained.

While previous studies frequently showed that *ompK36* mutations reduce fitness (51–55), it remains mostly unclear whether and how exactly this might be compensated. Ameliorating these costs may also play a crucial role in the emergence and maintenance of resistance within the bacterial population (68), even without antibiotic selection pressures. We thus performed a second EE experiment and evolved both 2003.2 and 2003.9 mutants (Fig. 1) to explore potential compensatory events that occur independently of antibiotic selection pressures. Since different growth conditions might directly affect evolutionary adaptation (69, 70), we used two complex media to increase the chance of identifying actual beneficial mutations. The first was LB medium, which contains no fermentable sugars and primarily provides amino acids as a carbon source (71). The second was cation-adjusted Mueller-Hinton broth 2 (MH-2), which is formulated for antimicrobial susceptibility testing according to EUCAST guidelines (42).

**FIG 4:**
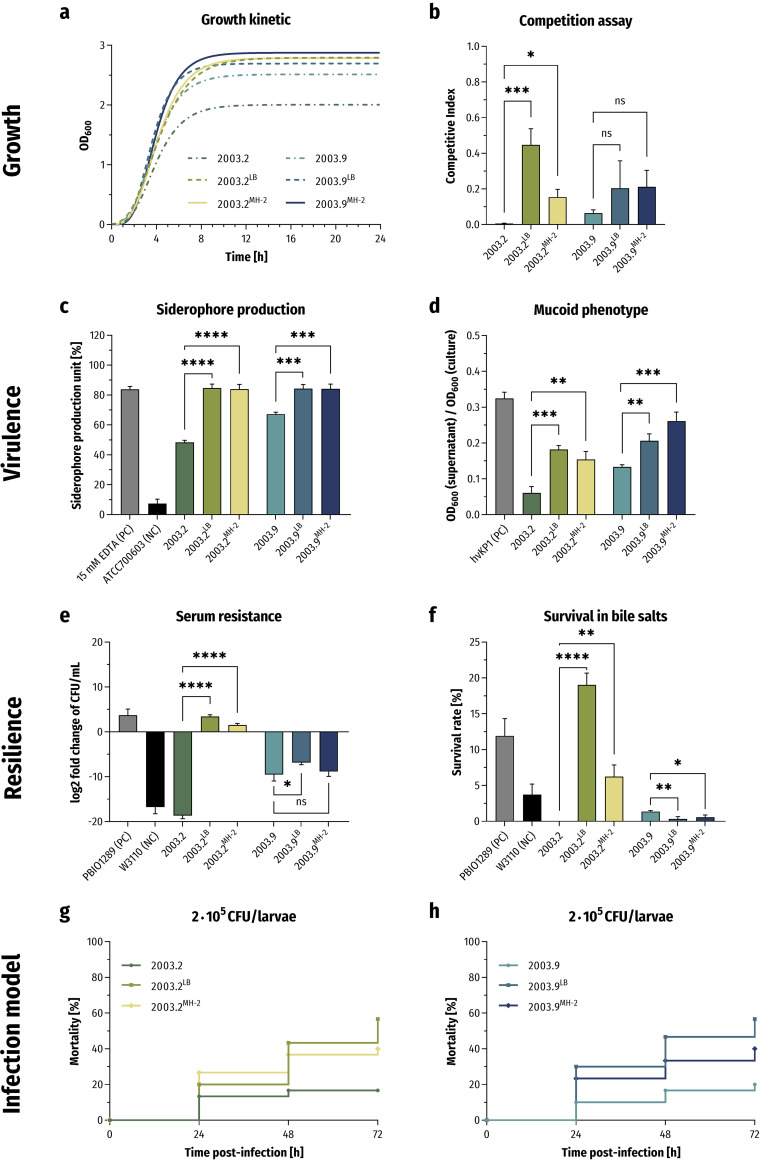
Phenotypic changes of the adapted variants of 2003.2 and 2003.9. (a) Gompertz growth-fitting curves of the growth kinetics in LB (*n* = 3). (b) Quantification of competitive interactions. The results are given as mean values and standard deviation of competitive indices (CIs). (c) The extent of secreted siderophore is presented as mean values of the siderophore production unit and standard deviation (*n* = 3). (d) Determination of mucoid phenotype by using a sedimentation assay (*n* = 3). The results are given as mean ratios of OD_600_ of supernatant after centrifugation at 1,000 × *g* for 5 min and total OD_600_ and standard deviation. (e) Survival in 50% human serum (*n* = 3). The results are given as mean values and standard deviation of log_2_ fold change of CFU/mL after 4 h of incubation in the presence of serum. (f) Resilience against 50 mg/mL bile salts (*n* = 3). The results are shown as mean percent survival rates and standard deviation. (g and h) Kaplan-Meier plot of mortality rates in the Galleria mellonella larvae infection model (*n* = 3). The results are given as mean percent mortality following injection of 2 × 10^5^ CFU/larvae. For all results, the adapted variants were compared to their respective parental strain using variance analyses (one-way ANOVA with Dunnett’s multiple comparison *post hoc* test); ns, not significant; *, *P* < 0.05; **, *P* < 0.01; ***, *P* < 0.001; ****, *P* < 0.0001.

During the second EE, we tracked the growth behaviors of each population every fifth day. Noticeably, after 15 days of serially passaging in LB and 25 days in MH-2, a fitness behavior of the evolved variants comparable to the growth of wild-type PBIO2003 was observed (Fig. 4a and b). Hence, compared to their parental strain 2003.2 (competitive index [CI] of 0.0060), the adapted variants 2003.2^LB^ (CI of 0.4480, *P* = 0.0002) and 2003.2^MH-2^ (CI of 0.1547, *P* = 0.0344) showed a significant increase in growth and competition. However, 2003.9^LB^ (CI of 0.2041, *P* = 0.2505) and 2003.9^MH-2^ (CI of 0.2117, *P* = 0.2230) exhibited only weak changes in their growth behaviors compared to the parental mutant 2003.9 (CI of 0.0647).

We hypothesized that fitness recovery is accompanied by an increase in virulence and resilience. We thus tested the adapted variants in our set of phenotypic experiments (Fig. 4c to f). Compared to the parental strain 2003.2, the adapted variants 2003.2^LB^ and 2003.2^MH-2^ showed a significant increase in siderophore secretion (Fig. 4c; 2003.2 48.3% and 2003.2^LB^ 84.7%, *P* < 0.0001; 2003.2^MH-2^ 83.9%, *P* < 0.0001) and mucoviscosity (Fig. 4d; 2003.2 0.061 and 2003.2^LB^ 0.182, *P* = 0.0003; 2003.2^MH-2^ 0.155, *P* = 0.0013). In addition, we observed a significant increase in resilience toward human serum and bile salts (Fig. 4e and f). In contrast, changes in the adapted variants 2003.9^LB^ and 2003.9^MH-2^ compared to their parent 2003.9 seemed slightly different. Again, we obtained a significant increase in siderophore production (Fig. 4c; 2003.9 67.3% and 2003.9^LB^ 84.4%, *P* = 0.0003; 2003.9^MH-2^ 84.2%, *P* = 0.0003) and mucoviscosity (Fig. 4d; 2003.9 0.134 and 2003.9^LB^ 0.207, *P* = 0.0044; 2003.9^MH-2^ 0.261, *P* = 0.0003), respectively. However, only 2003.9^LB^ showed a significant increase in serum resistance (Fig. 4e), and, interestingly, 2003.9^LB^ and 2003.9^MH-2^ showed a significant decrease in tolerance against bile salts (Fig. 4f) compared to 2003.9. Notably, the *G. mellonella* larvae infection model revealed increased mortality caused by each adapted variant originating from 2003.2 and 2003.9. By injecting 2 × 10^5^ CFU of 2003.2^LB^ and 2003.2^MH-2^ per larvae, respectively, we detected mortality rates of 20.0% (2003.2^LB^) and 26.7% (2003.2^MH-2^) after 24 h and 56.7% (2003.2^LB^) and 40.0% (2003.2^MH-2^) after 72 h of incubation (Fig. 4g). When using the same inoculation volume of 2003.9^LB^ and 2003.9^MH-2^ (Fig. 4h), we observed mortality rates of 30.0% (2003.9^LB^) and 23.3% (2003.9^MH-2^), resulting in overall mortality rates of 56.7% (2003.9^LB^) and 40.0% (2003.9^MH-2^) after 72 h.

In summary, with the second EE experiment, we obtained adapted variants that seemingly restored their fitness and virulence compared to the original wild-type strain. To assess the underlying mechanisms of these compensatory events, we next performed genomic analyses. When comparing the genomes of 2003.2^LB^ and 2003.2^MH-2^ with the parental strain 2003.2, our analysis revealed that the premature stop codon in *ompK36* and the read-through mutation of *dsbA* were still present. Moreover, we identified two different missense mutations in *rpoE* (2003.2^LB^: 154G>A [Glu52Lys]; 2003.2^MH-2^: 97G>T [Val33Phe]), encoding one of the primary regulators of the enveloped stress response system, which is activated through accumulated misfolded proteins and lipopolysaccharide (LPS) fragments in the periplasmatic space (72, 73). Upon alternative sigma factor E (σ^E^) triggering, it forms a holoenzyme with the bacterial core RNA polymerase complex (RNAP) and initiates transcription of more than 100 protein-coding genes, such as proteins required for DNA recombination and repair, lipid A biosynthesis, and LPS translocation as well as OMP membrane insertion (74, 75). Interestingly, both missense mutations were located in the highly conserved domain σ^E^_2_, which stabilizes the transient association of σ^E^ with the RNAP, thus forming the active holoenzyme and initiating transcription (76). Therefore, these *rpoE* mutations might destabilize the RNAP binding, resulting in decreased transcription levels of RpoE-regulated genes. Furthermore, this might reduce the ability of σ^E^ to compete with the other sigma factors for RNAP (77).

In contrast to the variants of 2003.2, when comparing the genomes of 2003.9^LB^ and 2003.9^MH-2^ with 2003.9, we only identified the expected deletion of transmembrane β-sheets between the loops L7 and L8 of *ompK36* but no other mutations.

Pathogenic bacteria rapidly respond to surrounding changes during different stages of infection by transcriptomic regulation (78, 79). These complex regulatory networks not only protect the cell from external stressors but also improve fitness and the expression of virulence-associated features such as enhanced iron uptake (79, 80). While costly genetic modifications in the *ompK36* gene in the presence of CAZ-AVI represent an advantageous trade-off, reduced membrane permeability in the absence of antibiotics itself is a stressor that resistant strains must cope with. This might be reflected by significant transcriptomic changes to compensate for fitness costs and overcome high cellular stress levels. This is why we next examined the transcriptomes of the adapted variants 2003.2^LB^, 2003.2^MH-2^, 2003.9^LB^, and 2003.9^MH-2^ compared with their parental strains 2003.2 and 2003.9, respectively (Fig. S1 to S3 in the supplemental material). The Clusters of Orthologous Groups (COG) analysis revealed an upregulation of genes encoding cell motility and extracellular structures in all adapted variants (Fig. S4 and S5 in the supplemental material). However, the adapted variants showed extensive downregulation of genes associated with information storage and processing, cellular processes and signaling, and metabolism. Interestingly, differential gene expression (DGE) analysis of 2003.2^LB^ and 2003.2^MH-2^ compared with 2003.2 revealed significant downregulation of genes whose transcription is induced by σ^E^, thus supporting our hypothesis that both missense mutations of *rpoE* resulted in a loss of function of this alternative sigma factor (Fig. S4 in the supplemental material). These downregulated σ^E^ regulon genes are required for proper folding and assembly of OMPs (*bamACDE*, *fkpA*, *skp*), phospholipid and LPS biogenesis and modification (*eptB*, *lpxP*, *phoP*), cellular processes and regulation (*csrD*, *cutC*, *htrA*, *rpoE*, *rseP*), as well as unknown functions (*yfeXY*) (75, 81). Furthermore, we noticed an upregulation of genes encoding porins (*ompA*, *ompK36*), which is usually associated with the inactivation or repression of σ^E^ during exponential growth (74). Taken together, these results strongly support the role of RpoE suppression in regaining fitness and virulence as shown by 2003.2^LB^ and 2003.2^MH-2^ results. Also note that we detected a significant increase of *ompK17* expression for both 2003.2^LB^ and 2003.2^MH-2^. Various studies reported that this small OMP, which is a structural homolog of OmpX of E. coli (82), is involved in cell adhesion and biofilm formation (83) and contributes to resistance against complement-mediated killing and *in vivo* mortality (84), thus possibly also participating in the restored phenotypes of 2003.2^LB^ and 2003.2^MH-2^.

The DGE analysis of 2003.9^LB^ and 2003.9^MH-2^ compared to their parental strain 2003.9 revealed a significant upregulation of *nhaA* (Fig. S5 in the supplemental material), which encodes an essential sodium proton antiporter involved in enhanced pH tolerance and sodium and volume homeostasis, crucial to cell viability (85). Interestingly, a previous study showed that the deletion of *nhaA* leads to severe attenuation of virulence in pathogenic E. coli strains in mammalian and avian infection models (86). Moreover, an orthologue of NhaA expressed by Yersinia pestis contributes to bacterial survival in the bloodstreams of infected mice (87), thus indicating that NhaA might contribute to fitness and virulence regain of 2003.9^LB^ and 2003.9^MH-2^. In addition, we detected a significant downregulation of *dksA*, an RNA polymerase-binding transcription factor, which is a part of the stringent response. Under nutrition-limited conditions, bacteria must adjust their metabolic activity and growth to maintain a balance between cell survival and proliferation. Therefore, they synthesize signaling nucleotides (ppGpp) that interact with two binding sites of RNAP, leading to transcription initiation. DksA binds to another binding site of RNAP and modulates its activity in conjunction with ppGpp (88). Additionally, in the absence of ppGpp, DksA regulates rRNA transcription initiation. A previous study demonstrated that the rRNA promoter activity of a Δ*dksA* mutant does not decrease following entry into the stationary phase, and the promoter does not respond to changes in growth rates or amino acid starvation (89). This might be another explanation for the fitness and virulence recovery of 2003.9^LB^ and 2003.9^MH-2^.

Finally, we verified that the resistant phenotypes were still present in 2003.2^LB^, 2003.2^MH-2^, 2003.9^LB^, and 2003.9^MH-2^ by again examining the MIC of a large panel of relevant antibiotic drugs and combinations (Table S1). Our analysis revealed that most resistance profiles of 2003.9^LB^ and 2003.9^MH-2^ strains were similar to their parental strain 2003.9. In contrast, we detected a difference regarding the last-resort antibiotic colistin in 2003.2^LB^ and 2003.2^MH-2^ (from 16 μg/mL to 0.5 μg/mL). It is known that incorporation of phosphoethanolamine and 4-amino-4-deoxy-l-arabinose in lipid A lowers the negative charge of the bacterial LPS, which subsequently leads to resistance (90). Note that here, the aforementioned decrease of RpoE activity in 2003.2^LB^ and 2003.2^MH-2^ leads to reduced expression of genes related to these lipid A modifications (*eptB*, *lpxP*, *phoP*), resulting in exposure of the negative charge and consequent colistin susceptibility.

In summary, here we show that a resistance-accompanying decrease in fitness and virulence was compensated following bacterial evolution in the absence of antibiotic selection pressure, while CAZ-AVI resistance was still present. Our genomic and transcriptomic analyses suggest that mutations in *rpoE* and expression differences of RpoE-regulated genes were drivers of this compensation along with, however, a loss of colistin resistance due to alterations of LPS.

## DISCUSSION

Although combinations of synergistically interacting drugs (such as combinations of β-lactams and β-lactamase inhibitors) are often used to treat infections, Hegreness et al. (91) showed that these combinations did not sufficiently suppress resistance acquisition. The authors concluded that mutations confer simultaneous resistance against both compounds. In particular, sublethal antimicrobial concentrations hereby appear to induce rapid mutagenesis (92). Starting with a subinhibitory concentration and daily increasing CAZ-AVI concentrations, we obtained two different CAZ-AVI-resistant mutants within 14 days only. Although this approach does not exactly reflect a guideline-oriented therapy in clinical practice (93), the rapid resistance development is remarkable and underlines the clinical importance of our finding.

As mentioned before, combining ATM with CAZ-AVI to treat bacterial pathogens that harbor both MBL and non-MBL is a last-resort drug possibility. Since a fixed-dose product of ATM-AVI is currently unavailable, increased tolerance of pathogens against this combination has been rarely described clinically (94). Quite recently, Nordmann et al. (95) reported the occurrence of E. coli strains showing phenotypic nonsensitivity against ATM-AVI, however, conferred by modifications of PBP3 with cooccurrence of different β-lactamases. Our study provides evidence of mutations in *ompK36* following EE of a clinical XDR K. pneumoniae strain, resulting in both resistance against CAZ-AVI and nonsensitivity against CAV-AVI with ATM. We show that these mutations resulted phenotypically in nonexpression of OmpK36 (2003.2, premature stop codon) and reduced pore diameter (2003.9, deletion of transmembrane β-sheets between the loops L7 and L8), respectively. Previous studies associated alterations of OmpK35/36 channels with CAZ-AVI resistance in K. pneumoniae in clinical settings (96–98), but, due to the complexities in underlying genetic mechanisms, interactions of specific β-lactamases and the diversity of *ompK35/36* variants, their potency in resistance contribution remains controversial (99). For example, Pagès et al. (100) demonstrated that ESBL-producing K. pneumoniae, which additionally lacked one or both OmpK porins, had increased MIC values of CAZ but were still susceptible to CAZ-AVI. Hence, the authors concluded that OmpK35 and OmpK36 did not contribute to the intracellular uptake of AVI through the outer membrane. In contrast, we unequivocally verified that the combination of missing OmpK35 expression and changes in the OmpK36 channel led to almost entirely reduced uptake of the third-generation cephalosporin CAZ and the bridged 1,6-diazabicyclo[3.2.1]octan-7-one derivative AVI. Interestingly, this seems to be independent of the expression of OmpA, as 2003.9 showed similar or sometimes higher relative uptake reductions, although we detected that OmpA was expressed. Moreover, the similarity of MIC changes and reduction in compound uptake indicates that the expression of OmpK36 with reduced pore diameter is as effective as the complete deletion of the porin. Results of another study support this finding (55). Consistent with the findings of our study, the authors demonstrated that two mutation types of OmpK36 had comparable potency in the contribution of β-lactam resistance. Furthermore, OmpK35 was shown to play only a minor role in this resistance propagation; an issue that we were not able to investigate more closely due to the intrinsic absence of OmpK35 in the wild-type strain PBIO2003. Overall, our results demonstrate the importance of OmpK36 in rapid resistance acquisition.

It is well-known and has been frequently described that resistance acquisition is, directly and indirectly, accompanied by a reduction of bacterial fitness and virulence (101). In fact, our porin-mutated strains 2003.2 and 2003.9 showed significantly decreased growth behaviors compared to wild-type PBIO2003. Moreover, we found a strong decrease in virulence-associated features and stress resilience, as also demonstrated by reduced *in vivo* mortality. Consistent with our findings, previous studies have reported a fitness reduction and lack of virulence of strains that obtain OMP alterations (51–55), thus indicating the key role of porins in maintaining fitness and consequently virulence. Overall, our results suggest that antibiotic resistance acquisition is a double-edged sword. On the one side, it is necessary for survival in environments with respective selection pressures. On the other side, resistance acquisition comes at a cost and may lead to reduced fitness, virulence, resilience, and mortality, likely resulting in decreased competition potentials and a lack of features required for the successful adaptation to new niches and pathogenicity. Interestingly, our genomic analysis showed that the costly membrane modifications were likely not compensated by a plasmid loss. Thus, the persistence of several large plasmids under high cellular stress conditions suggests that plasmid carriage does not inevitably reduce bacterial fitness (1, 102, 103).

Several pandemic high-risk clonal lineages have been identified in recent years, which successfully combine high-level virulence and resilience (1, 103–107) with extensive drug resistance, even in more pristine environments with low antibiotic selection pressures (108–110). This combination and the nonreversibility of resistance could be due to different compensatory events that counteract fitness costs (111). However, because of the broad phylogenetic diversity of bacterial hosts and the vast spectrum of compensatory mechanisms, reports and predictions of underlying adaptive processes that occur in clinical strains remain mostly unavailable. To address these issues, we performed a second EE analysis to track adaptational changes throughout the evolutionary process. We showed that the fitness costs of 2003.2 and 2003.9 were nearly completely ameliorated when evolved in two different media for 15 days (LB) and 25 days (MH-2). This rapid adaptation to external stress associated with fitness recovery might contribute to important changes in transmission dynamics and patient prognosis during infection (112). In addition, the fitness increase was accompanied by enhanced virulence levels of 2003.2^LB^, 2003.2^MH-2^, 2003.9^LB^, and 2003.9^MH-2^. While we identified mutations in *rpoE* and subsequent changes in expression levels of genes regulated by RpoE in the genomes of 2003.2^LB^ and 2003.2^MH-2^, our DGE analysis of 2003.9^LB^ and 2003.9^MH-2^ indicated the sole contribution of transcriptomic changes in the recovery of fitness and virulence. Note that the compensated fitness costs in the adapted variants 2003.2^LB^ and 2003.2^MH-2^ were accompanied by an increased susceptibility to colistin demonstrating collateral sensitivity, which might be a promising prospective therapeutic approach for treating chronic infections caused by XDR pathogens (49).

One limitation of our study is the “random” nature of mutations and transcriptomic and phenotypic changes, which might present differently upon repetition of the experiment. Moreover, during (experimental) evolution, numerous factors, such as variations in population size and selection pressure (113), might directly influence adaptational events. However, this does not diminish the significance of our findings but underlines the diversity of resistance evolution, compensation, and underlying processes. Another limitation is the possibility that mutations and changes in the mutants could be media adaptations that are difficult to correlate with natural circumstances (69). We cannot completely exclude this point without doubt. However, as can be seen in the DGE analysis, similar genes were up- or downregulated regardless of whether the second EE was performed in LB or MH-2 medium. The increased fitness and virulence of 2003.2^LB^, 2003.2^MH-2^, 2003.9^LB^, and 2003.9^MH-2^ was thus presumably not (only) based on media adaptations. Finally, to conclusively explain the underlying mechanisms behind the phenotypes would require depletion and/or complementation studies. Therefore, additional investigations will have to address these points further. Also, it might be promising to evaluate the impact of outer membrane modifications on the protective properties of promising vaccines whose immunization response is based on OmpK36 (114–116).

### Conclusion.

In this study, we not only highlight the important role of OmpK36 regarding β-lactam and AVI uptake as well as its contribution to resistance acquisition but also demonstrate the disadvantageous effects of changes in OMPs on bacterial fitness and virulence. More importantly, we show that fitness costs accompanied by this resistance acquisition were compensated rapidly and that bacterial virulence was almost completely restored, enabled by particular genomic and transcriptomic adaptations that involved major bacterial regulators.

## MATERIALS AND METHODS

### Bacterial strains and experimental evolution.

Bacterial strains used in this study are listed in Table 1. All strains were stored at −80°C in LB (Carl Roth, Karlsruhe, Germany) supplemented with 20% (vol/vol) glycerol (anhydrous; Merck, Darmstadt, Germany). Before use, one single colony from fresh overnight cultures on LB agar plates was individually inoculated in 5 mL of LB and grown under shaking conditions (130 rpm) at 37°C overnight.

**TABLE 1:** Overview of bacterial strains used in this study

Strain	ST	Relevant characteristics or genotype	References
Klebsiella pneumoniae			
PBIO2003	307	Wild-type strain (rectal swab, human), ancestral strain of 2003.2 and 2003.9 *bla*_OXA-48_, *bla*_CTX-M-15_, *bla*_SHV-106_, *bla*_TEM-1B_, *aac(3)-IIa*, *aph(3”)-Ib*, *aph(6)-Ib*, *fosA*, *tet34*, *sul2*, *mdfA*, *oqxAB*	1, 13
2003.2	307	*ompK36* (Gln146*) mutated variant of PBIO2003 (first EE) Parental strain of 2003.2^LB^ and 2003.2^MH-2^	This study
2003.2^LB^	307	In LB-adapted variant of 2003.2 (second EE, 15 days)	This study
2003.2^MH-2^	307	In MH-2-adapted variant of 2003.2 (second EE, 25 days)	This study
2003.9	307	*ompK36* (Δ78 bp; 972–1,049/1,125 nt) mutated variant of PBIO2003 (first EE) Parental strain of 2003.9^LB^ and 2003.9^MH-2^	This study
2003.9^LB^	307	In LB-adapted variant of 2003.9 (second EE, 15 days)	This study
2003.9^MH-2^	307	In MH-2-adapted variant of 2003.9 (second EE, 25 days)	This study
ATCC 700603	489	Laboratory reference strain (urine, human), negative control for siderophore secretion assay	143
hvKP1	86	Archetypal hypervirulent K. pneumoniae isolate (blood and liver, human), positive control for hypermucoviscosity assay	27, 144
Escherichia coli			
PBIO1289 (IMT10740)	1159	Internal reference APEC strain (environment, poultry), positive control for serum resistance and survival in bile salts	108, 145
W3110	10	Laboratory reference strain, negative control for serum resistance and survival in bile salts	146

To study CAZ-AVI resistance acquisition and increase the probability of receiving resistant representatives (117), we used 12 randomly chosen single colonies as individual biological replicates of PBIO2003 for our first EE approach (Fig. 1). The single colonies were inoculated in 1.5-mL tubes (Carl Roth, Karlsruhe, Germany) containing 1 mL of cation-adjusted Mueller-Hinton broth 2 (MH-2; Sigma-Aldrich, St. Louis, MO, USA) and grown at 37°C and 130 rpm. Following overnight incubation, 10 μL of bacterial precultures were transferred into a 96-well microtiter plate containing 190 μL MH-2 supplemented with CAZ-AVI (Zavicefta, Pfizer, New York City, NY, USA), resulting in a final concentration of 0.125/0.03mg/mL CAZ-AVI. The inoculated microtiter plates were incubated at 37°C without agitation for 24 h to allow “fixation” of mutations that occurred in the later growth phase (118). Daily, 10 μL of the culture was transferred in 190 μL of MH-2 with increasing concentrations of CAZ-AVI until some replicates tolerated concentrations of 16/4 μg/mL of CAZ-AVI. This is considered resistant according to the guidelines of EUCAST (42).

We then used a second EE approach to investigate compensatory events overcoming (putative) fitness burdens. One population, each of 2003.2 and 2003.9, was propagated in 5 mL of LB or MH-2 at 37°C and 130 rpm by transferring 5 μL of stationary-phase culture into fresh medium every day. We collected samples from the populations every fifth day and stored them at −80°C until further examination.

### Whole-genome sequencing.

One randomly chosen single colony was cultured overnight in MH-2 supplemented with 16/4 μg/mL CAZ-AVI. Total DNA was extracted using the MasterPure DNA purification kit for blood, version 2 (Lucigen, Middleton, WI, USA), according to the manufacturer’s instructions. Isolated DNA was purity controlled and quantified using a NanoDrop 2000 and Qubit 4 fluorometer (Thermo Fisher Scientific, Waltham, MA, USA), respectively. The DNA was shipped to the Microbial Genome Sequencing Center (MiGS; Pittsburgh, PA, USA) and, following library preparation, sequenced using 2 × 150 bp paired-end reads (Illumina NextSeq 550).

Raw sequencing reads of the parental strain PBIO2003 were processed as described before (1). The trimmed and filtered reads were mapped against the complete reference genome of the closely related strain PBIO1953 with breseq v.0.36.0 (119). Mutations evidenced by read alignments were applied to this reference with the help of the GenomeDiff tools shipped with breseq. This new reference of PBIO2003 was used for mapping sequencing reads of strains obtained through experimental evolution with breseq and deducing mutations.

Raw sequencing reads of evolved mutants were adapter-trimmed, contaminant-filtered, and quality-trimmed with BBDuk from BBTools v.38.89 (http://sourceforge.net/projects/bbmap/). FastQC v.0.11.9 (http://www.bioinformatics.babraham.ac.uk/projects/fastqc/) was used for quality control of both raw and trimmed reads. *De novo* genome assemblies at a maximum coverage of 100× were performed using shovill v.1.1.0 (https://github.com/tseemann/shovill) with SPAdes v.3.15.0 (120). Draft genomes were additionally polished outside the shovill pipeline by mapping trimmed reads to the contigs of the draft assemblies using BWA v.0.7.17 (121), and after processing of SAM/BAM files (sorting, marking of duplicates) with SAMtools v.1.11 (122), variants were called with Pilon v.1.23 (123).

### RNA isolation and sequencing.

The overnight cultures were set to 0.5 McFarland standard turbidity. Then, 3 mL of these bacterial suspensions were added to 27 mL of LB (10-fold dilution) and incubated at 37°C and 130 rpm until the optical density at *λ* = 600 nm (OD_600_) reached 0.2 turbidity (early log phase). Next, 1 mL of bacterial cultures was harvested, cooled down in liquid nitrogen for 5 s to inhibit further transcriptomic activity, and centrifuged (16,000 × *g* for 3 min at 2°C). Finally, the supernatants were discarded entirely, and the pellets were frozen in liquid nitrogen for 2 s and stored at −20°C (not more than 6 h) until further preparation. According to the manufacturer’s instructions, the total RNA was extracted using the RNeasy Mini Kit (Qiagen, Hilden, Germany). Isolated RNA was purity-controlled and quantified using a Qubit 4 fluorometer. The RNA was shipped frozen to LGC (LGC Genomics, Berlin, Germany) and, following rRNA depletion and mRNA library preparation, sequenced using 1 × 75 bp reads (Illumina NextSeq 550, nonstranded).

First, Trim Galore v.0.6.7 (https://github.com/FelixKrueger/TrimGalore) was used for the adapter and quality trimming of the raw sequencing reads. Next, the trimmed reads were mapped with Bowtie 2 v.2.4.4 (124) (mode: --very-sensitive-local) using the assembly of PBIO2003 as reference. Subsequently, gene counts were calculated using featureCounts v.2.0.1 (125) based on the annotation of PBIO2003. Finally, the count table was imported into R v.4.1.1 (https://www.R-project.org/), and differentially expressed genes were called with DESeq2 v.1.33.5 (126) in default mode, with one exception; genes with rowSums of <10 in the count table were removed before the analysis. We used an absolute 1.5 log_2_ fold change threshold with an adjusted *P* value lower than 0.01 to determine differences in gene expression (127). We excluded one replicate of 2003.9^LB^ due to a shift on principal component 1 (PC1) and PC2 obtained by visual evaluation of the plotted principal-component analysis (PCA; Fig. S1 in the supplemental material). All differentially expressed genes were compared to the COG (Clusters of Orthologous Groups of proteins) database v.2020 (128, 129) using Diamond v.2.0.11.149 (130) (mode: blastP, E value of ≤10^5^).

### Molecular modeling of OmpK36 protein structure.

To investigate the molecular effects caused by mutations of *ompK36*, we predicted the protein structure of OmpK36 by homology modeling. The initial model for the trimeric full-length OmpK36 construct was built within the multiple sequence viewer application in Maestro v.2020-4 (Schrödinger, New York, NY, USA). In total, five models were calculated based on the template structure of Protein Data Bank (PDB) number 6RCP (54) using a knowledge-based approach with side chain optimizations. A high-quality, clash-free model was selected and subsequently prepared with the protein preparation wizard (131) in Maestro to add hydrogens, assign bond orders, and optimize protonation state, followed by a short restraint minimization. In order to obtain the structure for 2003.9, the residues Lys324 to Asp350 were simply deleted in all three monomers, and terminal residues were capped with acetyl groups at the N termini and protected *N*-methyl-amide at the C-terminal ends. CHARMM force field parameter and protein standard files (PSF) for NAMD v.2.13 (132) were prepared using CHARMM-GUI (133). Finally, hydrogen mass repartition was applied using an in-house script that enables a timestep of 4 fs (134). All stages were simulated *in vacuo* with a cutoff for nonbonded interactions at 1.6 nm, including a switching function with a 0.1-nm region. After initial minimization for 50,000 steps, the system was simulated for 4 ns NVT (constant number of particles, volume, and temperature) at 310 K. Temperature was controlled by a Langevin thermostat with a 1ps^−1^ damping coefficient. The hydrogen bonds of β-sheet-forming residues were restrained using extra bonds with a force constant of 10.0 kcal/(mol × Å^2^). Both ends of the open elliptical β-barrel were slowly contracted with harmonic potentials of 0.5 kcal/(mol × Å^2^) in all three monomers simultaneously to close the region of segment deletion. Another trimeric homology was calculated from the 2003.9 sequence based on the final simulation snapshot as a template to rebuild the remaining loops with Prime (135).

### Identification of outer membrane proteins by MALDI-TOF MS.

The bacterial suspensions were set to 0.5 McFarland standard turbidity in deionized water, pelleted (13,800 × *g* for 5 min at room temperature [rt]), and resuspended in 300 μL of deionized water. Then, 900 μL of 99.8% (vol/vol) ethanol (Carl Roth, Karlsruhe, Germany) was added. Following two centrifugation steps (9,600 × *g* for 2 min at rt) and air drying (4 to 6 min), the pellets were resuspended in 20 μL of 70% (vol/vol) formic acid (Thermo Fisher Scientific, Waltham, MA, USA). The suspensions were mixed with 20 μL of acetonitrile (Carl Roth, Karlsruhe, Germany) and centrifuged (9,600 × *g* for 2 min at rt). Next, 1 μL of supernatants was spotted on a MALDI target plate (MBT Biotarget 96, Bruker Daltonik, Bremen, Germany) and left to dry at rt, and 1 μL of 130 μM 2,5-dihydroxybenzoic acid matrix (Sigma-Aldrich, St. Louis, MO, USA) was added onto each protein-containing spot. The extracted membrane proteins were analyzed by MALDI-TOF MS using a Microflex smart instrument (mass range of 2 to 40 kDa, laser intensity of 70%, number and frequency of shots of 200; Bruker Daltonik, Bremen, Germany). The analysis of spectra was performed using flexAnalysis software v.3.3 (Bruker Daltonik, Bremen, Germany).

### Cellular uptake of CAZ, AVI, and ATM.

The cellular uptake of the β-lactam antibiotics ATM (dissolved in water: dimethylformamide [DMF; 1:1]; Acros Organics, Geel, Belgium) and CAZ (dissolved in 0.1 M NaOH; Acros Organics, Geel, Belgium) and the non-β-lactam β-lactamase inhibitor AVI (dissolved in water; BioVision, Milpitas, CA, USA) was investigated by determining the increased cellular content of sulfur in terms of carbon monosulfide (*λ* = 258.0330 nm) based on uptake of the sulfurous compounds by using high-resolution continuum-source molecular absorption spectrometry (HR CS MAS) based on the graphite furnace technique. This investigation refers to experiments described previously (136) with minor modifications. First, overnight cultures were set to an OD_600_ of 0.3 turbidity (approximately 1.5 × 10^8^ CFU/mL). Then, 1 mL of these bacterial suspensions was transferred into 1.5-mL tubes and pelleted (4,000 × *g* for 10 min at rt). The supernatant was carefully discarded, and the pellets were resuspended in 1 mL of phosphate-buffered saline (PBS). Next, 100 μM sulfurous compound was added, and cells were incubated at 37°C without agitation. Samples containing only the solvent served as blanks. After 1 h of incubation, the cells were pelleted (4,000 × *g* for 10 min at rt) and washed once with 1 mL of PBS, and the collected pellets were stored at −20°C until further analysis.

For analysis, the pellets were thawed, resuspended in deionized water, and lysed by using a sonotrode (20 s, 9 cycles, 80 to 85% power; Bandelin Sonoplus, Berlin, Germany). An aliquot of the samples was spiked appropriately with the respective antibiotic and used for quantification. The measurements were performed with a contrAA 700 spectrometer (Analytik Jena, Jena, Germany). Ten microliters of the spiked samples was injected directly into pyrolytically coated graphite tubes (PIN-platform, Analytik Jena, Jena, Germany), followed by treatment applying for a time-temperature program as published before (137). Samples containing the lysate of bacteria were incubated only with the medium, and the solvent served as a blank. Thus, the endogenous sulfur content (blank) was subtracted from the absorbances when investigating the uptake of the sulfurous compounds. The mean integrated absorbances of 2 to 3 injections were used throughout the experiments. The uptake was expressed as a percentage of relative reduction related to PBIO2003.

### MIC.

Phenotypic antimicrobial susceptibility testing (AST) was performed using the Vitek 2 automated system (bioMérieux, Marcy l’Etoile, France). The MIC values of CAZ-AVI, CAZ-AVI with ATM, tigecycline (dissolved in dimethyl sulfoxide [DMSO]; Acros Organics, Geel, Belgium), and chloramphenicol (dissolved in 99.8% [vol/vol] ethanol; VWR International, Radnor, PA, USA) were determined by broth microdilution according to ISO standard 20776-1. Additionally, the MIC of colistin was examined using MICRONAUT MIC-Strip colistin (Merlin Diagnostika, Bornheim, Germany) according to the manufacturer’s instructions. All results were interpreted according to the published breakpoints and guidelines of EUCAST (42).

### Growth kinetics and competition.

Growth kinetics were assayed by continuously measuring the OD_600_. Briefly, overnight cultures were diluted 1:100 in 5 mL of fresh LB and incubated at 37°C and 130 rpm until the OD_600_ reached 0.5 McFarland standard turbidity. Then, the bacterial suspensions were diluted 10-fold, and 200 μL of cultures was transferred in triplicates into a 96-well microtiter plate (Nunc, Thermo Fisher Scientific, Waltham, MA, USA). Finally, the OD_600_ was recorded every 30 min using a microplate reader (FLUOstar Omega, BMG LABTECH, Ortenberg, Germany) at 37°C with 200 rpm orbital shaking.

The quantitative competition was determined by coincubation of PBIO2003 with 2003.2 and 2003.9 as well as 2003.2^LB^, 2003.2^MH-2^, 2003.9^LB^, and 2003.9^MH-2^. Here, the bacterial cultures were set to a 0.5 McFarland standard turbidity in LB as described for growth kinetics. Then, the reference (PBIO2003) and competitor strains were mixed in a 1:1 ratio by adding 100 μL of each strain to 20 mL of LB broth (approximately 1 × 10^5^ CFU/mL each). Twenty microliters of the resulting suspension was used for serial dilutions on LB agar plates (total CFU/mL) and LB agar plates containing 16/4 μg/mL CAZ/AVI (CFU/mL of competitor strain) to receive inoculum sizes. Next, the inoculated suspension was incubated at 130 rpm and 37°C for 24 h. Finally, the CFU/mL of the strains was again determined by performing serial dilutions. The competitive indices (CIs) were calculated as the output/input ratio of the competitor compared to the reference strain.

### Serum resistance and survival in bile salts.

Determination of survival in 50% human serum and 50 mg/mL bile salts (an equal mixture of cholic acid and deoxycholic acid) was performed as described previously (138), with minor modifications. Briefly, overnight cultures were diluted 1:100 in 5 mL of fresh LB and incubated at 37°C and 130 rpm until the OD_600_ reached 0.5 McFarland standard turbidity. Then, bacteria were pelleted (7,500 × *g* for 5 min at rt) and resuspended in 1 mL of PBS. One hundred microliters of sample was seeded in a 96-well microtiter plate containing 100 μL of human serum (United States origin, Sigma-Aldrich, St. Louis, MO, USA) or bile salts (100 mg/mL, dissolved in PBS; Sigma-Aldrich, St. Louis, MO, USA) per well (resulting in a final concentration of 50% human serum or 50 mg/mL bile salts and approximately 1 × 10^8^ CFU/mL). Next, 20 μL of each sample was collected, and the inoculum size was quantified by plating serial dilutions on LB agar plates incubated at 37°C overnight. The inoculated microtiter plates were incubated at 37°C without agitation for 4 h. After that, the number of survived CFU/mL was determined by plating serial dilutions and incubating at 37°C overnight. The positive control included in each experiment was the serum-resistant PBIO1289. The serum-sensitive W3110 served as the negative control. Serum resistance was expressed as log_2_ fold change of CFU/mL after treatment related to inoculum size. Percent survival in bile salts was obtained by determining differences in the CFU/mL after 4 h of incubation related to the inoculum count.

### Siderophore secretion.

The quantitative analysis of siderophore secretion was determined using a previously described method (24), with minor modifications. First, the bacterial cultures were set to 0.5 McFarland standard turbidity in 0.9% (wt/vol) NaCl solution. Then, 50 μL of bacterial suspension was added to 15-mL polypropylene tubes (Sarstedt, Nümbrecht, Germany) containing 5 mL of chelated M9 minimal salt medium (200 μM 2,2′-dipyridyl [Carl Roth, Karlsruhe, Germany] added M9 minimal salt medium [MP Biomedicals, Irvine, CA, USA]) supplemented with 2 mM MgSO_4_ (Carl Roth, Karlsruhe, Germany) and 0.3 % (wt/vol) of Casamino Acids (c-M9-CA [139]; BD, Franklin Lakes, NJ, USA). The strains were grown for 24 h at 37°C and 130 rpm. Next, 1 mL of bacterial cultures was collected in 1.5-mL tubes and centrifuged (4,900 × *g* for 20 min at rt), and 100 μL of siderophore-containing supernatant was transferred in triplicates to 96-well microtiter plates containing 100 μL of CAS shuttle solution (composited according to ref. 140). Additionally, fresh medium (blank) and 15 mM EDTA (positive control; Carl Roth, Karlsruhe, Germany) were included. The nonsiderophore producer ATCC 700603 served as a negative control. The mixtures were incubated in the dark for 30 min at rt, and the absorbance at a *λ* = 630 nm was measured using a microplate reader (CLARIOstar Plus, BMG LABTECH, Ortenberg, Germany). Secretion of siderophores was expressed as siderophore production unit in percent calculated as published previously (141).

### Hypermucoviscosity.

The hypermucoviscosity sedimentation assay was performed as described previously (60), with the following modifications. Again, the bacterial cultures were set to 0.5 McFarland standard turbidity in 0.9% (wt/vol) NaCl solution, and 50 μL of these bacterial suspensions was added to 5 mL of LB. Following an incubation period of 24 h at 37°C and 130 rpm, 1.5 mL of the cultures was collected in 2.0-mL tubes (Carl Roth, Karlsruhe, Germany) and centrifuged (1,000 × *g* for 5 min at rt). Two hundred microliters of the upper supernatant and 200 μL of the incubated culture were separately transferred each into triplicates to 96-well microtiter plates, and the OD_600_ was measured. The mucoid phenotype was expressed as the ratio of supernatant to total OD_600_.

### Infection of Galleria mellonella larvae.

The infections of larvae of the greater wax moth *G. mellonella* were performed as described previously (67). Briefly, 2 mL of overnight culture was collected and pelleted (16,000 × *g* for 5 min at rt). The pellets were washed once with PBS and diluted in PBS to an OD_600_ of 1.0 turbidity (approximately 2 × 10^9^ CFU/mL). The bacterial suspensions were further diluted to 2 × 10^6^ CFU/mL and 2 × 10^7^ CFU/mL, respectively. The larvae (proinsects, Minden, Germany) were randomly divided into groups with 10 individuals in each group, and 10 μL of the bacterial suspensions was injected in the right proleg. Additionally, one group of larvae was injected with 10 μL of PBS to ensure that death was not due to trauma from the injection. Each group was placed in 90-mm glass petri dishes kept at 37°C in the dark, and death was recorded every 24 h. Individuals were considered dead when they did not respond to physical stimuli and showed pigmentation. The results obtained with three independent assays were pooled for each strain to generate Kaplan-Meier plots of mortality rates (142).

### Statistical analysis.

Statistical analyses were performed using GraphPad Prism v.9.3.0 for Windows (GraphPad Software, San Diego, CA, USA). All phenotypic experiments were performed with three or more independent biological replicates. Unless otherwise specified, data were expressed as mean and standard deviation. Assessment of statistical significance was performed via analysis of variation (ANOVA) with Dunnett’s multiple comparison *post hoc* test. To analyze differences in cellular uptake of CAZ, AVI, and ATM, we used a one-sample *t* test. *P* values lower than 0.05 were used to show significant statistical differences among results.

### Ethics approval.

Ethical approval was given by the ethics committee of the University of Greifswald, Germany (BB 133/20). Informed patient consent was waived as samples were taken under a hospital surveillance framework for routine sampling. The research conformed to the principles of the Helsinki Declaration.

### Data availability.

The data for this study have been deposited in the European Nucleotide Archive (ENA) at EMBL-EBI under accession number PRJEB48690. Additional data of parental strains (PBIO1953 and PBIO2003) can be found under accession number PRJEB37933.
