# Supplementary material for: Extensively Drug-Resistant Klebsiella pneumoniae Counteracts Fitness and Virulence Costs That Accompanied Ceftazidime-Avibactam Resistance Acquisition
Source: Microbiol Spectr. 2022 Apr 18;10(3):e00148-22. doi: 10.1128/spectrum.00148-22 (PMC9241641; doi:10.1128/spectrum.00148-22)
Supplement: SUPPLEMENTAL FILE 1 — Supplemental material. Download spectrum.00148-22-s001.pdf, PDF file, 4 MB [file spectrum.00148-22-s001.pdf]

Supplementary Material

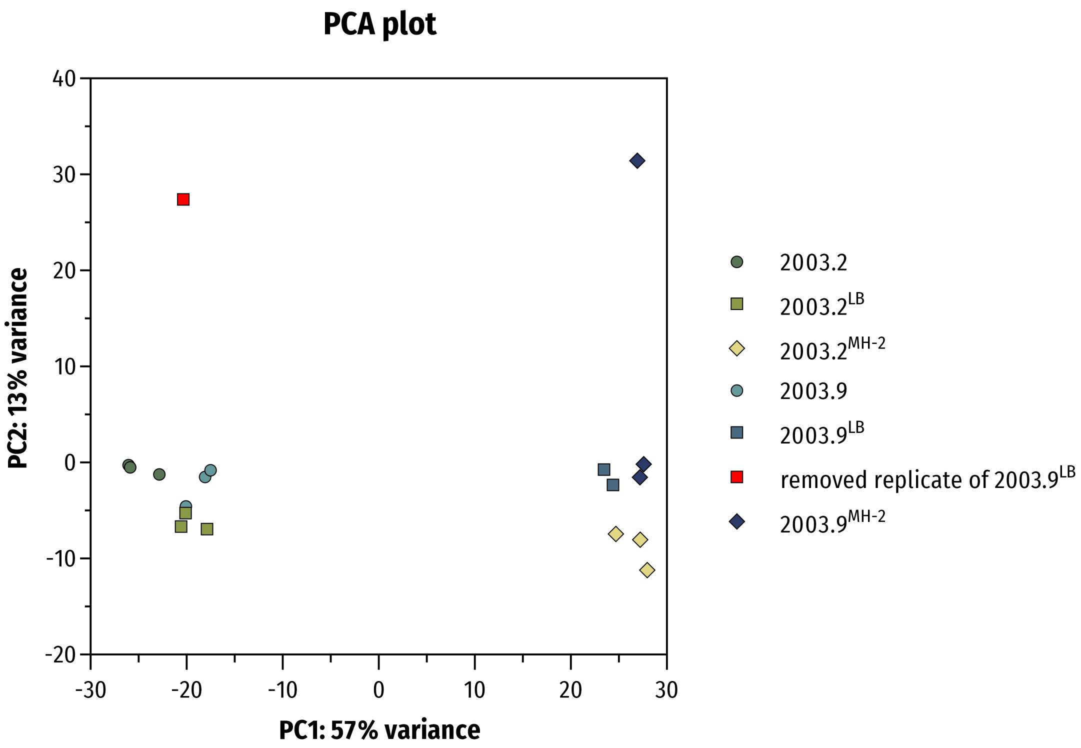

Supplementary Figure 1: Principal component analysis (PCA) of the RNA sequencing output. The PCA plot for the variance-stabilized transformation of the DESeq2 object was calculated on the basis of 1,000 top genes. We removed one replicate of 2003.9^{LB} (red) DESeq2 analysis because visualization revealed a shift on PC1 and PC2.

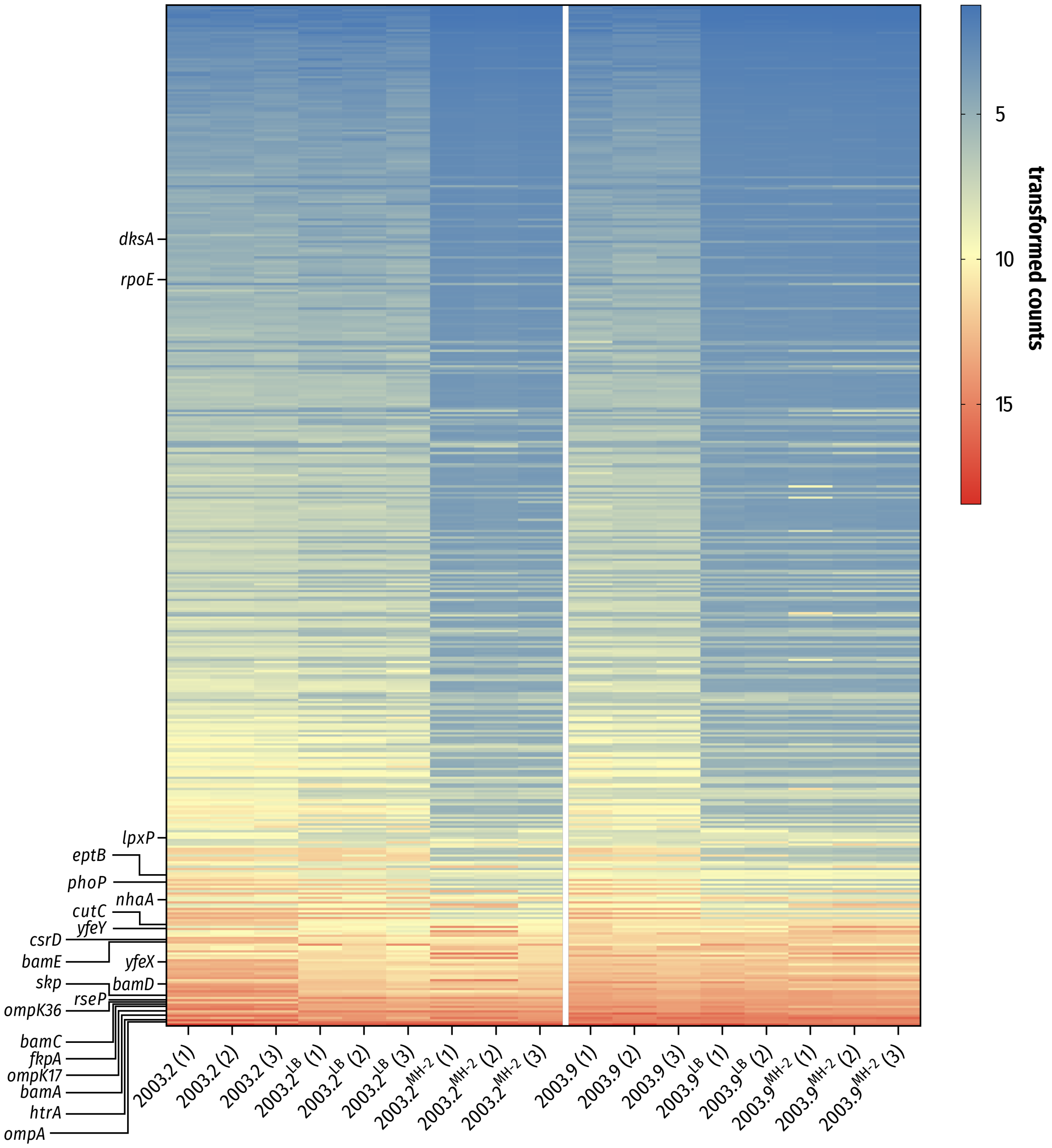

Supplementary Figure 2: Heatmap of differentially expressed genes (n = 459). Most important genes are shown on the left. The regularized log transformation function (rlog) from DESeq2 was used to convert counts to the log2 scale for better visualization.

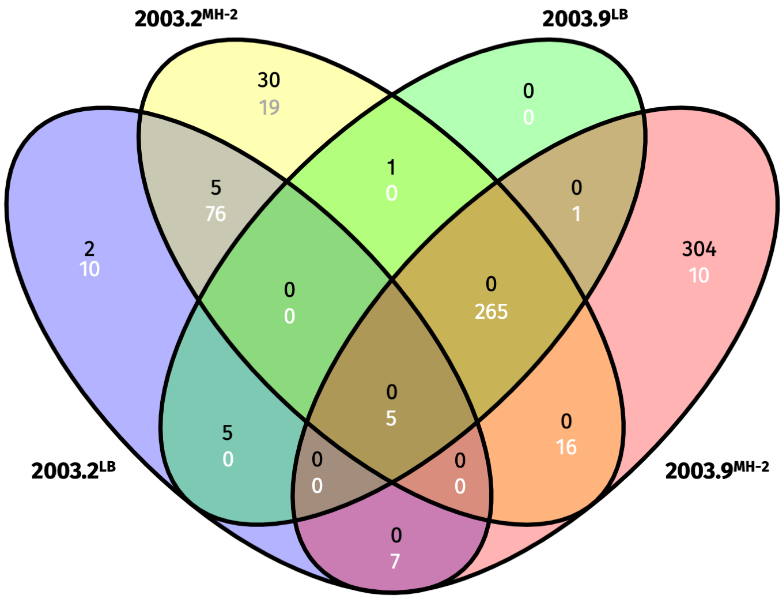

Supplementary Figure 3: Venn diagram of differentially expressed genes in 2003.2^{LB}, 2003.2$^{MH\text{-}2}$, 2003.9^{LB}, and 2003.9$^{MH\text{-}2}$. The Venn diagram shows the number of upregulated (black) and downregulated (white) genes in the four adapted variants. The visualization was created using ggvenn v.0.1.9.

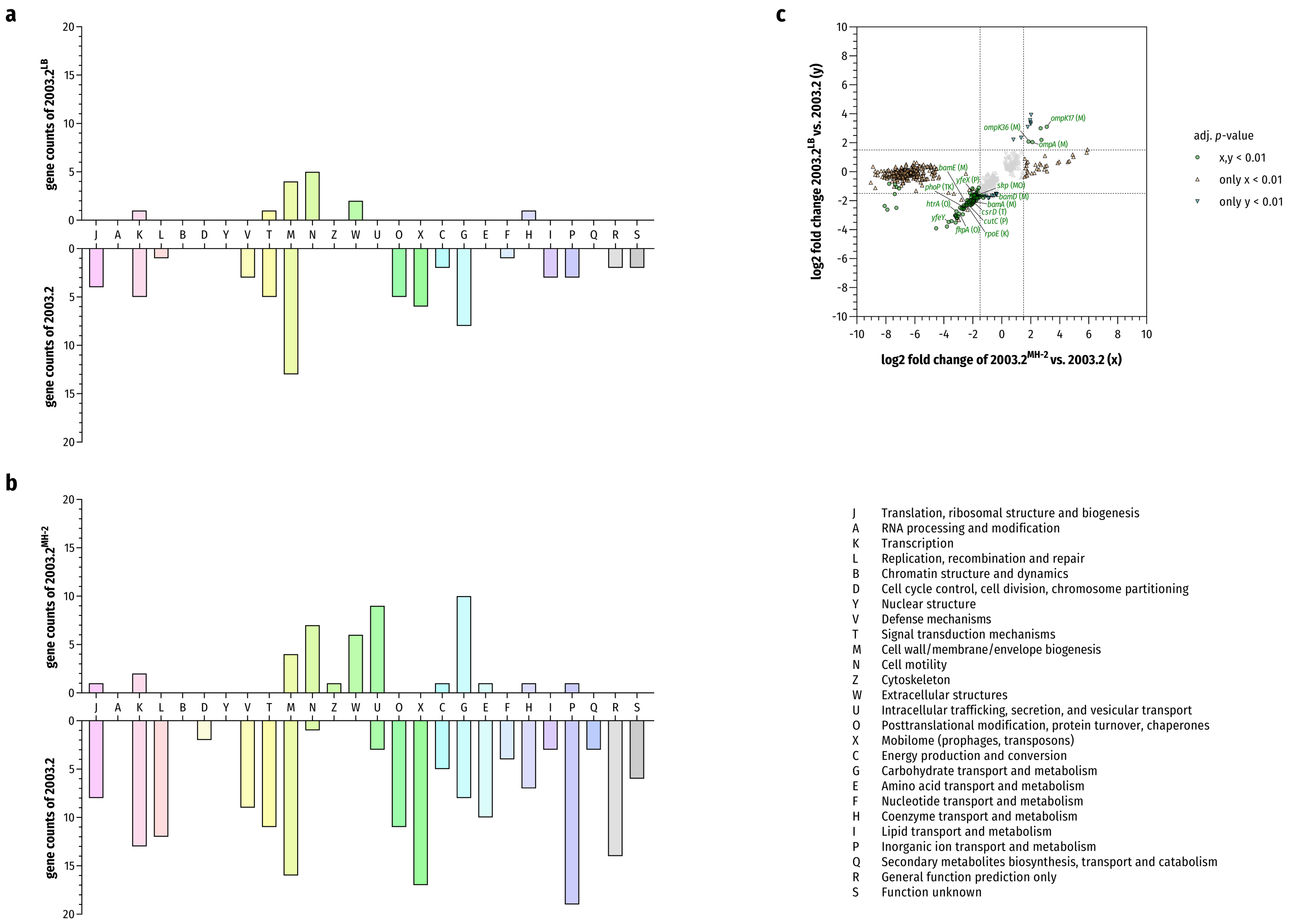

Supplementary Figure 4: Summary of the results of differential gene expression analysis of 2003.2, 2003.2^{LB}, and 2003.2^{MH-2}. a,b Functional classification of differentially expressed genes of 2003.2^{LB} (**a**) and 2003.2^{MH-2} (**b**) each compared to 2003.2 based on the Clusters of Orthologous Groups (COG) database. **c** Correlation analysis between 2003.2^{LB} (y-axis) and 2003.2^{MH-2} (x-axis). Similar gene expression profiles in the upper-right and lower-left corners indicate transcriptomic changes independent of the chosen broth medium. The most important genes are highlighted and classified according to COG.

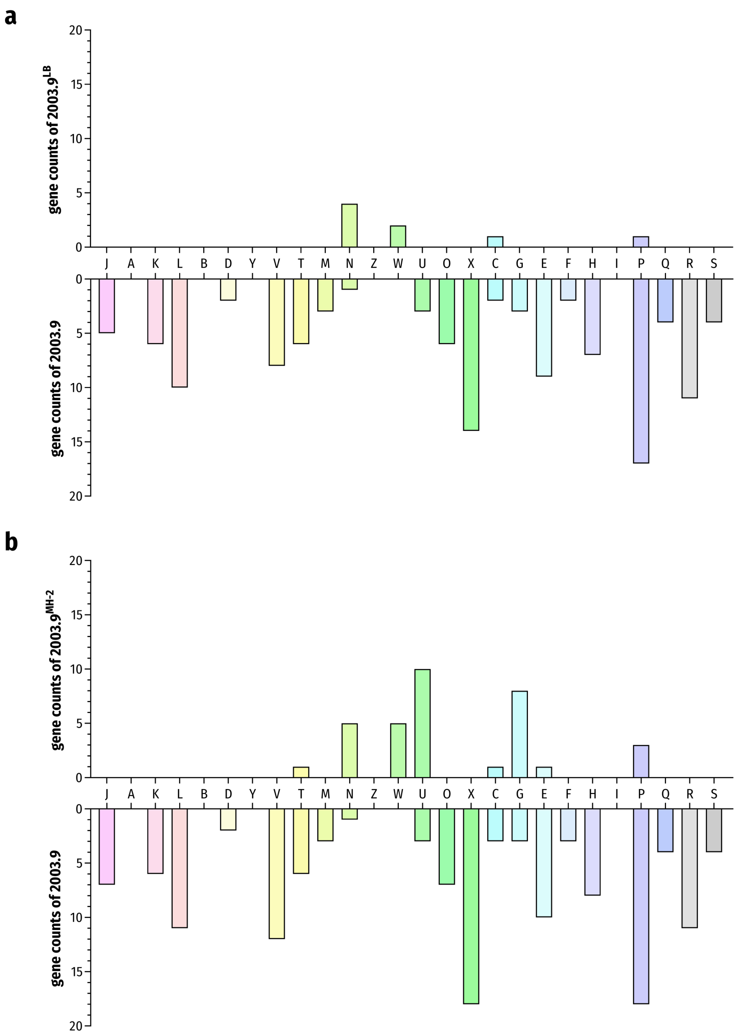

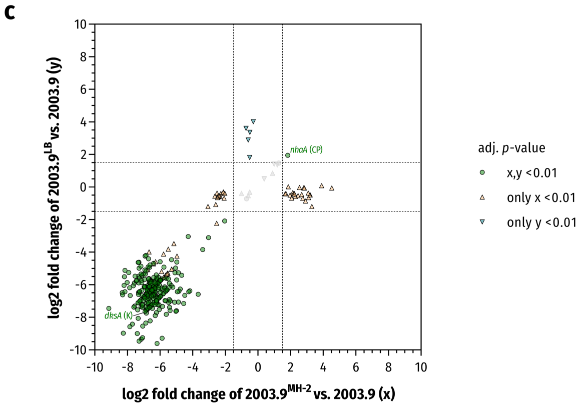

J Translation, ribosomal structure and biogenesis
A RNA processing and modification
K Transcription
L Replication, recombination and repair
B Chromatin structure and dynamics
D Cell cycle control, cell division, chromosome partitioning
Y Nuclear structure
V Defense mechanisms
T Signal transduction mechanisms
M Cell wall/membrane/envelope biogenesis
N Cell motility
Z Cytoskeleton
W Extracellular structures
U Intracellular trafficking, secretion, and vesicular transport
O Posttranslational modification, protein turnover, chaperones
X Mobilome (prophages, transposons)
C Energy production and conversion
G Carbohydrate transport and metabolism
E Amino acid transport and metabolism
F Nucleotide transport and metabolism
H Coenzyme transport and metabolism
I Lipid transport and metabolism
P Inorganic ion transport and metabolism
Q Secondary metabolites biosynthesis, transport and catabolism
R General function prediction only
S Function unknown

Supplementary Figure 5: Summary of the results of differential gene expression analysis of 2003.9, 2003.9^{LB}, and 2003.9^{MH-2}. a,b Functional classification of differentially expressed genes of 2003.9^{LB} (**a**) and 2003.9^{MH-2} (**b**) each compared to 2003.9 based on the Clusters of Orthologous Groups (COG) database. **c** Correlation analysis between 2003.9^{LB} (y-axis) and 2003.9^{MH-2} (x-axis). Similar gene expression profiles in the upper-right and lower-left corners indicate transcriptomic changes independent of the chosen broth medium. The most important genes are highlighted and classified according to COG.

Supplementary Table 1: Results of phenotypic antimicrobial susceptibility testing. [a] Interpretive categories according to EUCAST (The European Committee on Antimicrobial Susceptibility Testing 2021: Breakpoint tables for interpretation of MICs and zone diameters. Version 11.0.). [b] For susceptibility testing purposes, the concentration of the β-lactamase inhibitor is fixed at 4 mg/L. [c] Ceftazidime:aztreonam in the ratio 1:1 was used. MIC: minimum inhibitory concentration; n.a.: not applicable; R: resistant; S: susceptible.

	**PBIO2003 (wild-type)**		**2003.2**		**2003.2[LB]**		**2003.2[MH-2]**		**2003.9**		**2003.9[LB]**		**2003.9[MH-2]**	
**Antimicrobials**	**MIC [µg/mL]**	**S/R[a]**	**MIC [µg/mL]**	**S/R[a]**	**MIC [µg/mL]**	**S/R[a]**	**MIC [µg/mL]**	**S/R[a]**	**MIC [µg/mL]**	**S/R[a]**	**MIC [µg/mL]**	**S/R[a]**	**MIC [µg/mL]**	**S/R[a]**
Ampicillin	> 32	R	> 32	R	> 32	R	> 32	R	> 32	R	> 32	R	> 32	R
Ampicillin-sulbactam[b]	> 32	R	> 32	R	> 32	R	> 32	R	> 32	R	> 32	R	> 32	R
Piperacillin	> 128	R	> 128	R	> 128	R	> 128	R	> 128	R	> 128	R	> 128	R
Piperacillin-tazobactam[b]	> 128	R	> 128	R	> 128	R	> 128	R	> 128	R	> 128	R	> 128	R
Cefuroxime	> 64	R	> 64	R	> 64	R	> 64	R	> 64	R	> 64	R	> 64	R
Cefotaxime	> 64	R	> 64	R	> 64	R	> 64	R	> 64	R	> 64	R	> 64	R
Cefpodoxime	> 8	R	> 8	R	> 8	R	> 8	R	> 8	R	> 8	R	> 8	R
Ceftazidime	> 64	R	> 64	R	> 64	R	> 64	R	> 64	R	> 64	R	> 64	R
Ceftazidime-avibactam[b]	0.5	R	> 16	R	> 16	R	> 16	R	> 16	R	> 16	R	> 16	R
Aztreonam	> 128	R	> 128	R	> 128	R	> 128	R	> 128	R	> 128	R	> 128	R
Ceftazidime-avibactam-aztreonam[b,c]	> 0.0625/0.0625	n.a.	> 64/64	n.a.	> 64/64	n.a.	> 64/64	n.a.	> 64/64	n.a.	> 64/64	n.a.	> 64/64	n.a.
Ertapenem	> 8	R	> 8	R	> 8	R	> 8	R	> 8	R	> 8	R	> 8	R
Imipenem	> 16	R	> 16	R	> 16	R	> 16	R	> 16	R	> 16	R	> 16	R
Meropenem	> 16	R	> 16	R	> 16	R	> 16	R	> 16	R	> 16	R	> 16	R
Ciprofloxacin	> 4	R	> 4	R	> 4	R	> 4	R	> 4	R	> 4	R	> 4	R
Moxifloxacin	> 8	R	> 8	R	> 8	R	> 8	R	> 8	R	> 8	R	> 8	R
Gentamicin	> 16	R	> 16	R	> 16	R	> 16	R	> 16	R	> 16	R	> 16	R
Trimethoprim-sulfamethoxazole	≤ 20	S	≤ 20	S	≤ 20	S	≤ 20	S	≤ 20	S	≤ 20	S	≤ 20	S
Tigecycline	2	R	2	R	2	R	2	R	2	R	2	R	2	R
Colistin	16	R	16	R	0.5	S	0.5	S	16	R	16	R	16	R
Chloramphenicol	8	S	8	S	8	S	8	S	8	S	8	S	8	S
